# Mycotic aneurysm of the femoral artery complicating *Staphylococcus aureus *bacteremia: a case report

**DOI:** 10.1186/1757-1626-2-9386

**Published:** 2009-12-22

**Authors:** Patrícia Margarida Serra Carvalho, Joana Decq Mota, Patricia Gloria Dinis Dias, Antonio Oscar Carmona da Mota, Jose Julio Alves de Moura

**Affiliations:** 1 Serviço de Medicina II, Hospitais da Universidade Coimbra, Av. Bissaya Barreto e Praceta Prof. Mota Pinto, 3000-075, Coimbra, Portugal

## Abstract

**Introduction:**

*Staphylococcus aureus *is the major cause of bacteremia, with the potential for some complications, namely mycotic aneurysms, defined as irreversible dilatation of an artery due to destruction of the vessel wall by infection.

**Case presentation:**

The authors present the case of a 52 year-old-Caucasian male, admitted with *Staphylococcus aureus *bacteremia and mycotic aneurysm of the right superficial femoral artery, associated with advanced atherosclerotic process.

**Conclusion:**

Mycotic aneurysms are rare, and a high index of suspicion is needed, because appropriate treatment will certainly affect the outcome, as they are associated with high morbidity and mortality.

## Introduction

*Staphylococcus aureus *(SA) is the major cause of both community acquired and hospital acquired bacteremia and a possible source of diverse complications, namely metastatic seeding that can affect potentially any body site. Methicillin-resistant strains are now responsible for over a third of all staphylococcal bacteremias [1,2]. Humans are a natural reservoir of SA and rates of colonization are high among patients with type 1 diabetes, intravenous drug users, hemodialysis, surgical patients and with HIV infection [3]. This microorganism constitutes in some series the most common pathogen responsible for mycotic aneurysms [4-7].

Mycotic aneurysms are defined as a localized, irreversible dilatation of an artery due to destruction of the vessel wall by infection [8], which can arise following an infection of a previously healthy artery wall, or through secondary infection of a preexisting aneurysm. Despite unusual, mycotic aneurysms are seen more frequently in the femoral artery and abdominal aorta, and less frequently in the superior mesenteric, brachial, iliac and carotid arteries; cerebral circulation may also be affected [5]. Mycotic aneurysms are not exclusive of SA bacteremia and some other microorganisms may be implicated: *Salmonella*, *Streptococcus pneumoniae*, *Mycobacterium tuberculosis *and a long list of fungi, gram negative and gram positive bacteria that have been reported less frequently. However, since the introduction of antibiotics the bacteriology of infected aneurysms has changed from *Salmonella *and *Treponema species *to SA and gram negative bacilli [8].

There are a number of possible pathogenic mechanisms implicated in the formation of a mycotic aneurysm: septic emboli which occlude the vasa vasorum (frequently with origin in bacterial endocarditis), contiguous infective focus extending to the arterial wall, direct bacterial inoculation or bacteremic seeding of an existing intimal injury or atherosclerotic plaque [8,9].

## Case presentation

The authors present the case of a 52-year-old-Caucasian male, admitted to the emergency department in May 2007 with fever, polyarthralgia and peripheral edema, with inflammatory signs in the back of the left foot. Six days prior he had been discharged from the emergency department with the diagnosis of erysipelas, medicated with levofloxacin. Blood cultures were drawn. As his complaints persisted, he was readmitted and, because the blood cultures were revealed positive for methicillin-resistant *Staphylococcus aureus *(MRSA), he was admitted to an internal medicine ward with the diagnosis of MRSA bacteremia.

About three weeks earlier, he had been hospitalized in a Pneumology ward with the diagnosis of aggravated chronic obstructive pulmonary disease. During the hospital stay he developed phlebitis with abscess of the right arm at the site of an intravascular catheter. He was medicated with flucloxacilin with resolution. One day after being discharged he started fever.

In what refers to his past habits, he had an alcohol consumption quantified in 60 grams/day and was a heavy smoker - approximately 72 units of pack-years. In the last 8 years he had been a stoker, referring contact with black dust, without any protection of the airways.

His past history was marked by bronchitis in childhood and arterial hypertension; he referred two past episodes of bronchopneumonia. He was medicated with perindopril 5 mg, aminophilin 225 mg, furosemide 40 mg and inhaled bronchodilation therapy (tiotropium bromide and fluticasone/salmeterol).

He had family history of type 2 diabetes mellitus, cerebrovascular and coronary artery disease.

**Figure 1:**
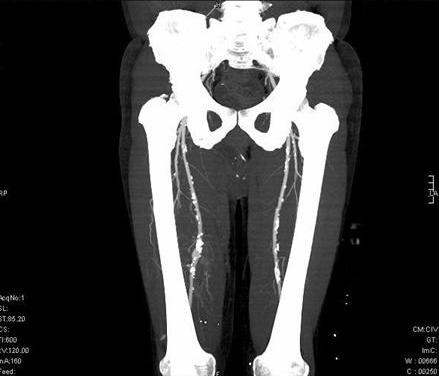
**CT scan**. Atheroma plaques in all arterial vascular beds focused (inferior limbs).

**Figure 2:**
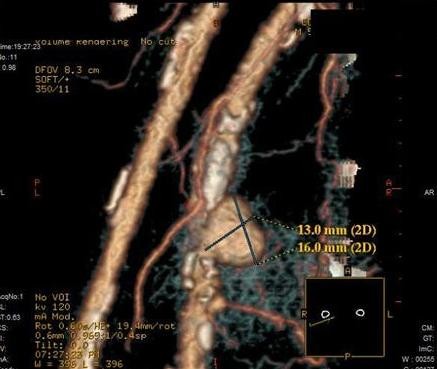
**Mycotic aneurysm**. CT scan with tridimensional reconstruction showing an aneurysmatic sacular formation in the dependence of the right superficial femoral artery.

**Figure 3:**
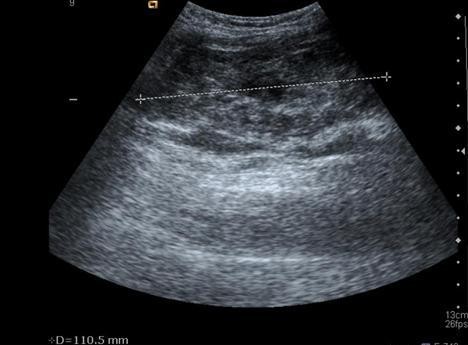
**Ultrasound**. Ultrasound showing an abscess with gas inside, located in the right thigh.

Blood tests on admission showed leukocytosis (16.5 G/L), elevated C reactive protein (17.21 mg/dl, with normal range < 0.5 mg/dl) and slight elevation of alanine aminotransferase (86 U/L, with normal range 10-40) and γ-glutamyl-transferase (84 U/L, with normal range 11-49). We started intravenous therapy with Trimethoprim-sulfamethoxazole 960 mg twice daily, as the antibiogram showed sensitivity for this antibiotic, and also for tetracyclines, rifampicin and glycopeptides. Initially, fever subsided, but in the sixth day his condition worsened, with fever and pain in his right thigh. Trimethoprim-sulfamethoxazole was suspended and he started intravenous vancomycin 1 g twice daily. The following day he presented a palpable, painful, non pulsatile mass with 3 cm in diameter located in the internal aspect of his right thigh, with edema, redness and heat; pedal pulse volume was diminished in his right limb; the ankle-brachial index was not determined; a femoral bruit could be heard; there were neither signs of hemorrhage nor clinical signs of carotid or renal artery stenosis. An ultrasound demonstrated soft tissue heterogeneity at the level of the superficial femoral artery, in a sagittal extension of 8.9 cm, corresponding to an atheromatous process of the artery associated with inflammation. An occlusion of the superficial femoral artery was detected by doppler and an angio-computed tomography (CT), multislice with tridimensional reconstruction demonstrated atheroma plaques in all arterial vascular beds focused (Figure 1), with lumen reduction above 70% in the medial third of the right superficial femoral artery, with an aneurysmatic sacular formation downstream (1.6 × 1.3 cm of antero-posterior axis) (Figure 2); a contiguous inflammatory process extending for 4 cm was noted. Surgical treatment was postponed until septic process was medically controlled. Rifampicin was added while continuing vancomycin. Seven days later, as the tenderness of the right thigh persisted, the ultrasound was repeated, showing an abscess with 11 cm in diameter, with gas inside (Figure 3). The following day the patient was submitted to surgical resection of a false mycotic aneurysm of the right superficial femoral artery, with placement of a femoro-femoral interposition graft of reversed homolateral internal saphenous vein. Metronidazole was started in association with vancomycin and rifampicin; at day 4 metronidazole and rifampicin were discontinued and replaced by imipenem because of a cutaneous allergic reaction. After fifteen days of vancomycin, creatinine started to rise (from basal value of 0.9 to 1.4 mg/dl, with normal range 0.7-1.3), and this antibiotic was replaced with linezolid 600 mg twice daily, intravenously.

**Figure 4:**
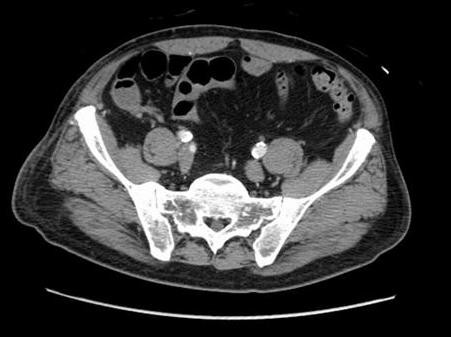
**Abdominal CT scan**. An angio CT scan showed calcic atheromatous plaques in the abdominal aorta and iliac arteries.

The patient underwent trans-thoracic echocardiographic study, which demonstrated fibrotic nodular thickening of the subvalvular aspect of the mitral valve and calcification of the head of the papillary muscles; a thickening of the intimate-media complex was obvious in the thoracic aorta, with multiple atheroma plaques, suggesting advanced atherosclerotic process. Later on, trans-esophageal echocardiogram was repeated with no evidence of infectious vegetations. The labeled leukocyte scintigraphy showed no other sites of accumulation besides the right thigh. A thoraco-abdomino-pelvic angio CT demonstrated calcified atheromatous plaques in the abdominal aorta and iliac arteries, with no evidence of other aneurysmatic formations (Figure 4).

In the twenty seventh day of admission, eighth day of imipenem and sixth day of linezolid, the fever completely subsided and the patient showed favorable progress. The only complication was a normocytic anemia in day 18 of admission (Hemoglobin - 7.4 g/dl, with MCV of 86.6 fL), with the need for packed red cells transfusion. Imipenem was stopped after nine days and linezolid after 28 days. He was discharged after 49 days to our outpatient clinic for follow up; 9 days after being discharged he underwent another transfusion for normocytic anemia (hemoglobin of 7.6 g/dl, with MCV of 84.8 fL). The white blood cell and the platelet counts remained unchanged; only red blood cell count was affected. He maintained smoking habits and, in spite of some claudicating complains he keeps full usage of his right limb.

## Discussion

SA is the leading cause of bacteremia and is often associated with secondary metastatic complications. Our patient had some risk factors for complications, namely infection by MRSA, with persistence of fever for more than 72 hours after admission [10]. He had risk factors for colonization by MRSA: prior hospitalization and treatment with antibiotics during his admission in a Pneumology ward; the nares are the primary reservoir for staphylococci, and individuals with nasal carriage of MRSA will also be colonized in areas of intact skin and other sites, for example sputum; our patient had COPD, which is associated with hypertrophy and hyperplasia of the mucus-secreting glands present in the epithelium of the airways, consequently with increased sputum production. A diagnosis at admission of skin or soft tissue infection also accounts as a risk factor for colonization; our patient had a diagnosis of erysipelas at admission. Colonization is associated with increased risk of subsequent MRSA infection with the same isolate [11,12].

Femoral aneurysms are often associated with percutaneous arterial access procedures or intravenous drug abuse [6,9], none of these present in this case. Besides not having history of arterial trauma, other risk factors that might predispose for mycotic aneurysms weren't present: bacterial endocarditis, local infection and impaired immunity (diabetes, alcoholism, corticosteroids, chemotherapy, malignancy and old age). The probable source of infection was the phlebitis associated with intravascular catheter during the prior admission. He had important cardiovascular risk factors (heavy smoker, hypertension, family history of type 2 diabetes), with advanced atherosclerotic process associated; the mechanism of infection was, most likely, the bacterial seeding of atherosclerotic plaques. Whether this was an infection of a previous aneurysm or a primary infection of the arterial wall, with formation of a new aneurysm we do not know.

The classic clinical presentation is that of a painful, pulsatile, enlarged mass, in a patient with systemic symptoms such as fever; hemorrhage or thrombosis can occur[6,9,13]. Our patient had a painful, non pulsatile mass, with inflammatory signs present and a local bruit; he was febrile and there were no signs of hemorrhage. The described signs are eventually masked by overlying inflammation and mycotic aneurysms may be misdiagnosed. The diagnosis is confirmed by imaging studies (ultrasound, computed tomography, angiography or magnetic resonance angiography) and, sometimes, mycotic aneurysms are only demonstrable in this way. Infected aneurysms are often associated with radiographic findings: the presence of air within the aneurysm, local inflammation, contained rupture or saccular or lobulated aneurysm [9]. In our patient the diagnosis was confirmed by angio-CT scan multislice with tridimensional reconstruction, which demonstrated a sacular formation, with a contiguous inflammatory process. Systemic features of inflammation and anemia are frequently present, and blood cultures are positive in 50-85% of cases[4] On admission our patient had elevated C reactive protein, leukocytosis and positive blood cultures; anemia wasn't present (hemoglobin - 14.5 g/dl).

Our patient was first treated with Trimethoprim-sulfamethoxazole, but after six days, as his condition worsened and because this antibiotic usually has less intrinsic activity against staphylococci, we chose to start vancomycin associated with rifampicin, with the goal of increasing bactericidal activity. Surgical treatment was initially postponed, first because there were no signs of hemorrhage, and second because primary repair isn't possible in infected areas, as it is associated with more severe complications, such as graft infection or anastomotic dehiscence [6,13,14]. However, despite medical treatment our patient developed an abscess and was submitted to resection of the aneurysm and revascularization procedure in day 16 of admission, without any further complications. Anaerobic bacteria were probably involved in the abscess formation, particularly with gas present; unfortunately, the collected material wasn't sent for culture. Arterial ligation with excision of the aneurysm, together with local debridement of all necrotic tissue and drainage of associated abscess remains the first treatment step; revascularization at the time of the resection of the aneurysm offers a better outcome for limb function and autogenous veins are more resistant to infection [6,13,14]. After surgery, broad spectrum antibiotics were started, with empiric coverage of gram negative and anaerobic bacteria; although admittedly empiric, some studies recommend 4-6 weeks of parenteral antibiotic therapy, with extension of treatment time if inflammatory biomarkers don't subside [8,9]; our patient was treated for almost 50 days, with complete resolution of the systemic features of inflammation. Linezolid replaced vancomycin (the patient developed acute renal failure), with good response, despite the occurrence of anemia after being discharged, probably due to linezolid therapy, as it can cause myelosuppression, related with the duration of treatment; in this case red blood cell line was the only one affected.

Mycotic aneurysms are associated with high morbidity and mortality rates, and combined therapy results in better outcome [4,15].

Our patient was anticoagulated with subcutaneous enoxaparin during admission, and then antiaggregated with acetylsalicylic acid after discharge; treatment with statin was started, with maintenance of anti-hypertensive drugs. He initiated a smoking cessation intervention program in the Pneumology outpatient clinic and started nicotine replacement with nicotine gums.

What is noticeable is that there was neither endocarditis nor any other foci of metastatic infection, and the mycotic aneurysm was the only complication.

In the follow up there was no recurrence of the infectious process and no acute ischemia of the limb, in spite of some claudication complaints. Cilostazol, with antiplatelet and vasodilating properties, indicated for symptomatic relief in cases of intermittent claudication, isn't commercially available in our country.

Notwithstanding medical advice, the patient maintains smoking habits, which can contribute to worsening of his peripheral arterial disease (PAD); smoking cessation is critical for delaying the progression of PAD, and also to reduce cardiovascular morbidity and mortality.

## Conclusion

Infected femoral aneurysms constitute a challenging problem in what concerns treatment options, as they are associated with high mortality and morbidity, including limb loss. A high index of suspicion should be present in our minds whenever an inflammatory mass appears near a large artery, so that prompt treatment is initiated.

## Consent

Written informed consent was obtained from the patient for publication of this case report and accompanying images. A copy of the written consent is available for review by the Editor-in-Chief of this journal.

## Competing interests

The authors declare that they have no competing interests.

## Authors' contributions

PC and AM were responsible for the diagnosis and medical treatment; PC and JM wrote the manuscript; PD, AM and JAM provided clinical and scientific orientation and were major contributors in writing the manuscript; all authors read and approved the final manuscript.
